# High Level of Pre-Treatment HIV-1 Drug Resistance and Its Association with HLA Class I-Mediated Restriction in the Pumwani Sex Worker Cohort

**DOI:** 10.3390/v14020273

**Published:** 2022-01-28

**Authors:** Rachel Willim, Elnaz Shadabi, Raghavan Sampathkumar, Lin Li, Robert Balshaw, Joshua Kimani, Francis A. Plummer, Ma Luo, Binhua Liang

**Affiliations:** 1 Max Rady College of Medicine, University of Manitoba, Winnipeg, MB R3E 3P5, Canada; willimr1@myumanitoba.ca; 2 Center for Biosecurity, Public Health Agency of Canada, Ottawa, ON K1A 0K9, Canada; elnaz.shadabi@phac-aspc.gc.ca; 3 Advanced Technology Platform Centre, Regional Centre for Biotechnology, Faridabad 121001, India; Raghavans1@gmail.com; 4 National Microbiology Laboratory, Public Health Agency of Canada, Winnipeg, MB R3E 3L5, Canada; Lin.li@phac-aspc.gc.ca (L.L.); Ma.Luo@phac-aspc.gc.ca (M.L.); 5 Data Sciences Platform, George & Fay Yee Centre for Healthcare Innovation, University of Manitoba, Winnipeg, MB R3E 0T6, Canada; Robert.Balshaw@umanitoba.ca; 6 Department of Medical Microbiology and Infectious Diseases, Rady Faculty of Health Sciences, University of Manitoba, Winnipeg, MB R3E 0J9, Canada; jkimani@csrtkenya.org (J.K.); umliangb@gmail.com (F.A.P.); 7 Department of Medical Microbiology, University of Nairobi, Nairobi 00100, Kenya; 8 Kenya AIDS Control Project, University of Nairobi, Nairobi 00100, Kenya; 9 Department of Biochemistry and Medical Genetics, Rady Faculty of Health Sciences, University of Manitoba, Winnipeg, MB R3E 0J9, Canada

**Keywords:** human immunodeficiency virus, antiretroviral therapy, drug-resistant mutation, human leukocyte antigen, next-generation sequencing technology

## Abstract

Background: We analyzed the prevalence of pre-antiretroviral therapy (ART) drug resistance mutations (DRMs) in a Kenyan population. We also examined whether host HLA class I genes influence the development of pre-ART DRMs. Methods: The HIV-1 proviral DNAs were amplified from blood samples of 266 ART-naïve women from the Pumwani Sex Worker cohort of Nairobi, Kenya using a nested PCR method. The amplified HIV genomes were sequenced using next-generation sequencing technology. The prevalence of pre-ART DRMs was investigated. Correlation studies were performed between HLA class I alleles and HIV-1 DRMs. Results: Ninety-eight percent of participants had at least one DRM, while 38% had at least one WHO surveillance DRM. M184I was the most prevalent clinically important variant, seen in 37% of participants. The DRMs conferring resistance to one or more integrase strand transfer inhibitors were also found in up to 10% of participants. Eighteen potentially relevant (*p* < 0.05) positive correlations were found between HLA class 1 alleles and HIV drug-resistant variants. Conclusions: High levels of HIV drug resistance were found in all classes of antiretroviral drugs included in the current first-line ART regimens in Africa. The development of DRMs may be influenced by host HLA class I-restricted immunity.

## 1. Introduction

Human immunodeficiency virus 1 (HIV-1) continues to be a major global health threat, with an estimated 37.6 million people living with HIV (PLWH) as of 2020 [1]. The Joint United Nations Programme of HIV/AIDs (UNAIDS) made an ambitious 90–90–90 goal of diagnosing 90% of those living with HIV, providing antiretroviral therapy (ART) to 90% of those who have been diagnosed and achieving suppressed viral load in 90% of PLWH on ART by 2020 [2]. Although the 90–90–90 target was not reached, the attempt led to unified efforts to increase testing, diagnosis, as well as effective treatment of PLWH [1]. These global efforts are necessary to reach the next UNAIDS sustainable development goal—95–95–95 by 2030 [3,4]. More recently, the Undetectable = Untransmittable (U = U) campaign has been launched [5]. The results of large studies on sexual transmission of HIV in thousands of serodiscordant couples showed that “individuals living with HIV who have an undetectable level of virus in their blood due to ART are unable to transmit the virus to others” [6,7,8].

One of the keys to achieving these goals is increased access and effectiveness of ART. However, wide use of ART has also led to rising rates of HIV drug-resistant mutations (DRMs) to a majority of the currently available drug classes, including the newer generations [9,10,11]. The HIV virus genome is incredibly variable due to its fast replication rate (around 10^8^ virions/day) and the error-prone RT, which introduces approximately 1 substitution error/genome/replication cycle [11]. Although the acquisition of HIV is usually from one viral variant, multiple variants (quasispecies) develop within the host over the subsequent weeks after infection [9]. Introduction and maintenance of HIV DRMs can naturally occur as viruses replicate under host immune pressure, through transmission from one host to another (transmitted drug resistance—TDR), or through selective pressure after exposure to antiretroviral drugs (acquired drug resistance—ADR) [10,12,13,14,15,16,17]. As such, pre-existing HIV DRMs (pre-ART DRMs) can be found in individuals who are yet to be exposed to ART. Pre-ART drug resistance is capable of compromising treatment efficacy and resulting in virologic failure evidenced by progressive clinical status, decreasing CD4 T-cells, and increasing viral load [17]. Routine monitoring of HIV patient populations for DRMs has been thus recommended by the World Health Organization (WHO). Low- and middle-income countries (LMIC) are increasing their collection of national data, as evidenced by the 57 additional countries initiating HIV DRM surveys from 2004 to 2020 [10]. Current guidelines in high-income countries recommend baseline genotypic testing for DRMs to nucleos(t)ide analogue reverse transcriptase inhibitors (NRTIs), non-nucleos(t)ide analogue reverse transcriptase inhibitors (NNRTIs), and protease inhibitors (PIs), reserving integrase strand transfer inhibitor (INSTI) DRM testing for those who have been previously exposed to that class of medication [18]. However, this strategy—increased reliance on population-wide testing data—may not be feasible in LMICs.

There were significant increases in the prevalence of pre-existing DRMs in all regions studied from 2001 to 2006 in LMICs, lining up with increases in antiretroviral (ARV) availability [17]. The highest levels of resistance in most regions have been linked to the NRTI and NNRTI drug classes [19,20]. The first-generation NNRTIs have a lower genetic barrier to resistance than others, resulting in higher levels of population resistance. The WHO thus recommended switching first-line therapy to dolutegravir (DTG), a second-generation INSTI medication [19]. DTG couples a higher genetic barrier of about three mutations necessary for resistance with high potency, making it a clinically efficacious medication. However, LMICs have limited access to DTG, which puts them at greater risk of increased population levels of HIV DRMs [4]. Additionally, low abundance drug-resistant variants (LADRVs; <20% of viral quasispecies) attracted researchers’ and clinicians’ attention after the launch of next-generation sequencing (NGS) technologies in 2005. Not only can NGS reliably detect LADRVs, but it can also perform large-scale sequencing, making it the ideal method for population-based HIV DRM surveillance. More importantly, pre-ART LADRVs were significantly associated with ART treatment failure in over 40% of studies, including individuals on first-line NNRTI-based regimens [20]. Cost-effective NGS methods are being developed to improve access in LMICs where high-efficiency sequencing would contribute to surveillance DRM data [10,19].

It is important for low resource settings to improve their HIV DRM data collection and analysis to assess population-wide trends regarding DRMs, including LADRVs, to which they can tailor their first-line therapy regimens [10,18]. The objectives of this study are: (1) to determine pre-treatment HIV-1 Drug Resistance in drug treatment naïve women in a Kenya Cohort and (2) to determine the influence of host HLA class I genes on the development of pre-treatment DRMs. We investigated the distributions of pre-ART DRMs in the *pol* region of HIV-1 proviral DNAs from ART-naïve women in Kenya using NGS technologies and explored the potential influence of host HLA class I genes on the development of pre-ART DRMs.

## 2. Methods

### 2.1. Study Participants and Samples

Study participants (*n* = 266) were all HIV-infected women enrolled between the years 1987 and 2010 in the Pumwani (a district of Nairobi) sex worker cohort established in Nairobi, Kenya. Because antiretroviral drug treatments were not available prior to 2003 when the PEPFAR was introduced, as well as the CD4 counts criteria for ART treatment, the participants did not receive antiretroviral therapy before 2003; participants enrolled later did not receive ART because their CD4 counts were above 200 cell/µL. The HIV+ blood samples, PBMCs, or buffy coats were collected from all participants at enrollment (*n* =266) or resurvey visits (*n* = 171). This study was approved by the Ethics Committee of the University of Manitoba and the Ethics and Research Committee of Kenyatta National Hospital, and written informed consent was obtained from all participants.

### 2.2. Amplification and Sequencing of HIV Genome

HIV-1 proviral DNA was amplified using a nested approach [21,22]. The first round of PCR amplified the full HIV genome using published primers MSF12b (HXB2 location 623–649) and ofm19 (HXB2 location 9632–9604) located in the 5′-LTR and 3′-LTR region using the Expand Long Template PCR System (Roche Diagnostics GmbH, Mannheim Germany) at the recommended conditions. Three sets of PCR primers were used for nested PCRs with Expand High Fidelity PCR System (Roche Diagnostics GmbH, Mannheim Germany) to generate 3 overlapping PCR products covering the full HIV-1 genome. This approach was used successfully to amplify full HIV-1 genomes from 300 samples [23]. The amplified PCR products were purified, quantified, and sequenced with Roche 454 GS FLX platform at 1000-fold coverage.

### 2.3. HLA Genotyping

A sequence-based high-resolution typing method [24,25] was used to genotype three HLA class I genes of all participants. DNA was isolated using QIAmp DNA Mini Kit and QIAgen EZ1 Blood Robot (QIAgen Inc., Mississauga, ON, Canada). HLA-A, -B, and -C genes were amplified by PCR with gene-specific primers [24,25]. The purified PCR products were sequenced with BigDye cycle sequencing kits (Applied Biosystems, Waltham, MA, USA) using sequence-specific primers and analyzed with an ABI3730 Prism Genetic Analyzer. HLA-A, -B, and -C alleles were assigned using CodonExpress^TM^, a computer software program developed based on a taxonomy-based sequencing analysis [24].

### 2.4. Sequence Analysis

The raw reads generated from the Roche 454 were aligned to HXB2, and only the sequencing reads mapped to the *pol* region of the HXB2 genome (coordinates: 2294–5096; 2803 bps) were extracted for HIV DRM identification and for consensus sequence generation using HyDRA Web (http://hydra.canada.ca; 13 July 2020). Default settings were selected, and reads were filtered to have a minimum variant quality score of Q30 and 100 bp length. The mutations were determined based on the standard mutation database. The minimum amino acid (AA) frequency for variant calling was 1%, the minimum read depth was 100, and the minimum mutation count was 5: an error rate of 0.0021 was used. In order to reduce the intrinsic error rate of the Roche 454 [18], only variants with a minimum Reference 12 is missing. Kindly include this and re-order the references to ensure that they run chronologically. 2% frequency were selected for analysis. The consensus sequence for each sample was generated using a threshold of 20%. The generated consensuses of protease (PR), reverse transcriptase (RT), and integrase (IN) of each sample were determined for HIV subtypes using Recombinant Identification Program (RIP) at HIV Sequence Database site (https://www.hiv.lanl.gov/content/sequence/RIP/RIP.html; 25 October 2017). The identified DRMs were cross-referenced with the 2009 WHO HIV surveillance mutations, Stanford University HIV Drug Resistance Database (Stanford HIV DB), and IAS-USA 2019 Drug Resistance Mutations Update [25,26]. The level of drug resistance was determined to be high, intermediate, or low using the Stanford HIV DB [25]. A patient-specific database was generated with patient IDs, characteristics including age, country of origin, CD4 counts, HIV subtype, and HIV stage (if available). Each DRM identified per patient was added to the table and merged in the case of multiple samples for one patient. If a patient had the same DRM at two different collection times, the mutation was counted once to portray the number of people with each variant accurately.

### 2.5. Statistical Analysis

Data analysis was conducted using IBM^®^ SPSS^®^ Statistics for Macintosh, V27.0.1.0 (IBM Corp., Armonk, NY, USA) [27]. HLA alleles and HIV DRMs present in more than 1% of participants/samples were included in our analysis. Fisher’s exact test was used to examine associations between individual DRMs and HLA class I alleles. Associations with *p*-values less than 0.05 were considered potentially relevant and were extracted for further characterization. The false discovery rate was controlled at 0.05 to adjust *p* values for multiple comparisons using the Benjamin–Hochberg method. Kaplan–Meier survival analysis was carried out to estimate the effect of HLA-restricted DRMs on time taken for CD4 + T cell to decline to below 200 cells/µL (diagnosis of AIDS). The log-rank test was used to compare the time of CD4+ T cell declining to <200 cells/µL between two groups: participants harboring HLA-specific DRMs vs. participants without HLA-specific DRMs. A *p*-value less than 0.05 was considered significant.

## 3. Results

### 3.1. Characteristics of the Study Participants

A total of 266 HIV+ women enrolled in the Pumwani cohort were included in the study; among them, 126 (47%) were from different regions of Kenya, 123 (46%) from Tanzania, and 14 (5%) from Uganda. Those from Tanzania and Uganda were from areas around Lake Victoria. Most of the women were Bantu speakers (~93%), and a small number of them were Nilote speakers. None of them had received ART. Participants ranged in age from 18 to 54 years, with a median (Q1, Q3) age of the participants of 35 (25, 45). The median duration since HIV-1 diagnosis was 7.67 years. The dominant HIV-1 subtype in *pol* gene was A1 (*n* = 150, 56.4%), followed by D (*n* = 25, 9.4%) and C (*n* = 14, 5.3%); HIV-1 subtype B was rare (*n* = 1, 0.4%). The average CD4 count was 295 cells/μL. The characteristics of the study participants are summarized in Table 1.

### 3.2. Pre-ART Drug Resistance Mutations

Among all participants, 98% of them had at least one DRM with frequency ≥2% within the viral population, including mutations at highly polymorphic sites and potential APOBEC mediated mutations. The total number of DRMs in each patient ranged from 1 to 15, with a mean of 6. A total of 58 unique variant positions were identified; 25 (43%) of these were in the reverse transcriptase (RT) region, 18 (31%) were in the protease (PR) region, and 15 (26%) were in the integrase (IN) region. Of those 58 mutations, 42 (72%) were determined to be non-polymorphic and unlikely to be APOBEC mediated [25]. One hundred and thirty-eight individuals (52%) had one or more DRM conferring at least potentially low-level resistance to one of the currently available ARV medications (according to Stanford HIV DB [20]) and 42% of participants harbored one or more DRM to one of the current first-line ARV options used in Kenya and surrounding countries (Table 2) [28,29]. One hundred and one participants (38%) harbored at least 1 WHO surveillance DRM (SDRM) with a range of 1–4 SDRMs/per patient.

Ninety-one percent of the participants harbored an NNRTI-associated variant, making it the drug class with DRMs in the highest number of participants. This was followed by PRs (61%), NRTIs (45%), and INSTIs (40%). Variants conferring resistance to one of the available ARVs (based on Stanford HIV DB or listed as a WHO SDRM [25,26]) were considered to be clinically important variants (Table 2).

**Figure 1:**
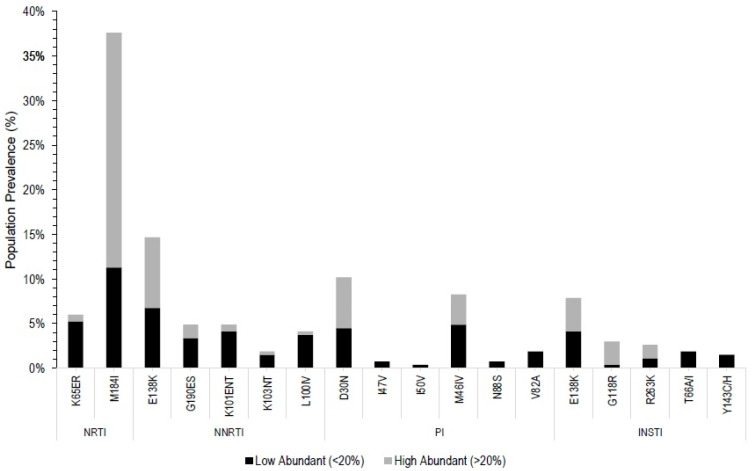
Pre-ART HIV DRM frequency divided by antiretroviral drug classes. Pre-ART DRMs were classified into low (dark color) or high (light color) frequency variants according to the portion of variants in the viral population (quasispecies) of each sample. Low abundant variant: the proportion of variant was <20% of the quasispecies; high abundant variant: the proportion of variant was >20% of the quasispecies. Only the clinically significant DRMs are shown. NRTI: M184I, K65ER; NNRTI: E138AK, K101ENT, G190ES, L100IV, K103NT; PI: D30N, M46I, V82A, N88S, I47V, I50V; INSTI: E138K, G118R, R263K, T66IA, Y143CH. DRMs: drug resistance mutations. NRTI: nucleos(t)ide reverse transcriptase inhibitor. NNRTI: non-nucleos(t)ide reverse transcriptase inhibitor. PI: protease inhibitor. INSTI: integrase strand transfer inhibitor.

The most prevalent clinically important mutations for each drug class are as follows with the study sample prevalence: M184I (37%) (NRTI), E138AK (15%) (NNRTI), D30N (10%) (PI), and E138K (8%) (INSTI) (Figure 1). The prevalence of these DRMs is very similar among the participants from different countries (Supplementary Table S1). The most prevalent single mutation overall, regardless of clinical importance, was K103E (RT), with a prevalence of 77% (result not shown). K103E frequently co-occurred with K103R, which had a prevalence of 61%. Fifty-eight percent of the study population had both the K103E and K103R variants.

The drug with the highest levels of resistance was Lamivudine (3TC), with 41% of the participants having the M184I and/or K65ER mutations. Other first-line therapy options with population-level resistance were Tenofovir (TDF), Efavirenz (EFV), Nevirapine (NVP), Lopinavir (LPV), Raltegravir (RAL), and Dolutegravir (DTG). The mutations conferring resistance to DTG, the newly introduced and WHO recommended INSTI, include E138K (IN), G118R, and R263K with prevalences of 6%, 3%, and 2%, respectively.

### 3.3. Pre-ART Drug Resistance Associated with HLA Class I Alleles

A total of 65 HLA Class I alleles and 29 HIV DRMs were observed in at least 1% of the study participants.

Of the 65 alleles, 23 (35%) were Class I A, 25 (38%) were Class I B, and 17 (26%) were Class I C. More than 1800 HLA-by-HIV DRM interactions were examined using Fisher’s exact test. We looked at the associations with the lowest uncorrected *p*-values, giving 53 potentially relevant associations (unadjusted *p* < 0.05). Fourteen of these 53 potential associations involved clinically important DRMs; their characteristics are summarized in Table 3.

**Figure 2:**
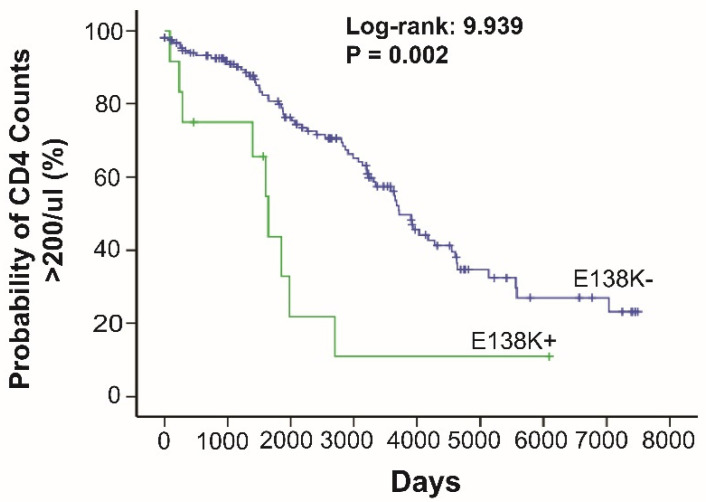
Kaplan–Meier survival analysis of RT E138K mutation on HIV disease progression to CD4+ T cells. Kaplan–Meier survival analysis was carried out to estimate the effect of HLA-specific adapted escape mutation-RT E138K on the time of CD4+ T cell counts to below 200 cells/µL (diagnosis of AIDS). Log-rank test was used to compare the time of CD4+ T cells declining to <200 cells/µL of two groups: the participants harboring RT E138K mutations and those who do not have the mutations. A *p*-value < 0.05 is considered significant. RT: reverse transcriptase.

The examination of the effect of the identified clinically important DRMs on the disease progression by Kaplan–Meier analysis showed that E138K_RT mutation is significantly associated with disease progression (*p* = 0.002) (Figure 2). Sub-Saharan countries with either a 5–10% or >10% HLA allele frequency in their population are listed in Table 3. The HLA–DRM associations involving HLA alleles at >10% frequency in Kenya are A*68:02 with DRMs G190ES (*p* = 0.008), IN E138K (*p* = 0.042), C*17:01 with DRMs M46I (*p* = 0.018), and IN E138K (*p* = 0.021). The most significant association was between the mutation T97A and A*66:01 (*p* = 6.20^−7^; *p* = 0.001 after correction for multiple tests), a high-frequency allele in both Kenya and Uganda.

We checked whether the same DRMs could be detected from samples collected at different years from the same study participants on the assumption that if the HLA class I restricted CTL response drive the development of a specific DRM, the DRM will be maintained in the participant without other factors such as ART. We observed such examples in our study. For example, E138K, a well-known escape mutation [30], was first identified in participant ML874 (A*68:02+) in the blood sample collected in 1996, and this DRM was also detected in the samples collected in 2003 from the same participant. Moreover, E138A was identified first in participant ML264 (A*68:02+), and in samples collected in 1996, the DRM was also present in two samples collected from the same patient more than 7 and 7.5 years later in 2003. Both patients have the A*68:02 allele that is associated with the DRM. These results support the assumption that HLA class I-restricted CTLs exert selective immune pressure on RT138 amino acid and maintain K138AK mutations in these individuals over the course of infection.

## 4. Discussion

Surprisingly, 98% of ART-naïve participants had at least one detectable HIV-1 variant, 38% of participants harbored at least one WHO SDRM, and 42% had at least a low-level resistance DRM to one of the first-line medications used in Kenya [28]. This is to be compared with previous studies reporting TDR prevalence of 8% in men who have sex with men (MSM) in Coastal Kenya from 2005 to 2017 [29], SDRM prevalence of 11% in four African countries from 2013 to 2019 [31], pre-treatment DRM prevalence of 24% in rural Kenya from 2008 to 2013 [32], and a 9.7% SDRM prevalence in Nigeria from 2013 to 2017 [33]. Importantly, resistance was identified to all major classes of antiretroviral drugs (NNRTIs, NRTIs, PIs, and INSTIs) currently used in Kenya (Table 2). The higher prevalence of pre-ART DRMs in our study is likely contributed by using NGS technology wherein sequence coverage of 1000x was reached compared to previous studies employing Sanger sequencing or OLA detection of a specific mutation. Eight (44%) of the clinically significant mutations (*n* = 18) were detected with frequency less than 10%, and one (5%) was present at a frequency between 10 and 20%. LADRVs (frequency < 20%) have been associated with an increased risk of virological failure in a dose-dependent manner with respect to mutant load, regardless of medication adherence [34,35,36]. Our results highlight the importance and utility of NGS and high coverage sequencing techniques [34,37,38].

RT K103E (77%) and K103R (61%) were the most common variants identified in our study. K103E, listed as a rare variant by Stanford HIV DB [25], can be selected by NNRTI medications but does not seem to confer any reduced susceptibility to them. When present with the variant V179D, which was not found in our participants, K103R can reduce susceptibility to NVP and EFV approximately 15-fold [25]. Knowledge of the high level of K103R polymorphism in this study population is important if population levels of the V179D variant increase. In previous studies, the K103 variant was shown to persist even in participants who tested negative for ARV drugs in their system, eliminating the possibility of acquisition of ARV medications illegitimately and suggesting there is alternative selective pressure maintaining that mutation [39].

The clinically significant mutations with the highest prevalence among the study participants were M184I (37%) and E138AK (15%) on RT, D30N (10%) and M46I (8%) on PR, and E138K (6%) on IN. Each of these variants confers at least low-level resistance to one or more of the currently recommended ARV drugs, except D30N, which confers resistance to NFV, a discontinued PI [20]. Slightly divergent from the guidelines, commonly used medications in Kenya and surrounding countries, as of 2020, include the combined regimens AZT + 3TC + NVP, TDF + 3TC + EFV, and AZT + 3TC + EFV [38]. Twenty-four participants (9%) harbored HIV-1 variants conferring low to high-level resistance to both EFV and NVP, while 41% had resistant mutations to 3TC. Of note, the presence of the M184I mutation in a patient’s quasispecies is not a contradiction to regimens containing 3TC or FTC. This is due to the finding that the M184I mutation decreases viral replication fitness and increases viral susceptibility to other ARV drugs TDF, dFT, and AZT [25]. M184 variants were identified in numerous studies within this region [23,37,38]. Interestingly, the M184V variant was not common (<1%, not shown) in this cohort. It is possible that the combination of M184I with other DRMS, especially E138AK, makes the isoleucine at position184 less likely to change to valine (to the more stable and fit valine variants [35]), as the M184I mutation exhibits high-level reduced susceptibility to FTC/STC. More importantly, 6% of the participants had the IN mutation, E138K, which confers low-level resistance to the newly recommended DTG or, when present with other IN variants, may synergistically lead to intermediate level resistance against DTG. A higher prevalence of IN-associated DRMs was seen in our study than in the others. The majority of studies in Kenya and surrounding countries have not yet included IN sequence data, and the ones that included IN sequence data did not find a high prevalence of INSTI DRMs [34,37,38]. In a recent study of a Congolese population, T97A, a variant conferring reduced susceptibility to INSTIs when acting synergistically with other INSTI mutations, was identified in 11% of the participants [25]. In our study, 12% of participants harbored the T97A variant (Table 3). R263K, a variant shown to be selected by DTG in vitro, was present in 2.3% of our sample with a mean quasispecies frequency of 34.9% (Figure 1), a startling prevalence considering that DTG is a relatively new drug and newer INSTIs have a high genetic barrier to resistance. Our results provide a rationale for increased surveillance of INSTI in this population and support the WHO’s recent recommendations [19].

HIV mutates rapidly to escape host immune responses, and host leukocyte antigen (HLA) class I-restricted CD8+ T cell responses represent a major selective pressure driving and shaping HIV evolution (or mutations) in the absence of ART within the HIV-1 positive population [13,15,40]. As such, pre-ART DRMs could be introduced and maintained by HLA-restricted immunity [18]. In our study, we examined associations between predominant HLA alleles and pre-existing DRMs. Among a total of 53 potentially relevant correlations identified, 14 of them involved clinically important DRMs, encompassing NNRTIs, PIs, and INSTIs, although most of these associations were very weak. Only the T97A variant showed a significant association with HLA*66:01 (adjusted *p* = 0.001) after correction for multiple tests. Failure to detect these associations is due to the reduced statistical power by the low prevalence of these specific HLA alleles and DRMs in the participants. Thus, the real association might be hidden. This is supported by the fact that E138K_RT mutation is associated with faster CD4 T cell decline by the Kaplan–Meier analysis (*p* = 0.002) (Figure 2). As E138K_RT is a well-known escape mutation [30], it suggests that the HLA class I restricted CD8 CTL responses that drove E138K to escape mutation. Indeed, the E138K_RT is significantly associated with several high-frequency HLA class I genotypes such as A*68:02 (17.99%), B*45:01(11%), and A*66:01(7.5%). High-frequency HLA alleles, with >10% allele frequency in the general Kenyan population associated with variants identified in our study, include A*68:02 and C*17:01. Both of these HLA class I alleles are associated with the INSTI related variant E138K (*p* = 0.048). E138K confers potentially low to intermediate level resistance to DTG alone and can act synergistically with other variants to confer greater resistance. A*66:01, a common allele in Kenya and Uganda, was strongly associated with the T97A variant (adjusted *p* = 0.001). As mentioned above, T97A can act synergistically with other INSTI mutations to reduce susceptibility to all INSTI medications. Participants with the aforementioned HLA Class I alleles are more likely to harbor these drug-resistant variants, increasing the risk of virologic failure after prescribing medications with potentially reduced efficacy. Most importantly, these HLA alleles are enriched in African populations such as the Ghanaian and South African black populations (Table 3). Evidently, pre-treatment HLA testing would provide patient-specific insight into potential DRMs. To our knowledge, this is the first study from Kenya to report HLA-associated HIV-1 variants on the RT, PR, and IN regions of *pol* in predominately non-B subtypes. Several studies found associations between HLA alleles and drug-resistant variants, mainly in subtype-B viruses from high-income countries [30,41]. More comparable to our study population, McCluskey et al. (2021) performed a study on individuals from Uganda and identified the INSTI variant L74I to be associated with HLA A*02, B*44:15, and C*04:07 in predominately subtype A1 viruses [42]. In our study, the L74I variant was associated with HLA B*15:01 (*p* = 0.011), an association also found in subtype B viruses in Switzerland and Australian cohorts [43]. Therefore, more studies are necessary to comprehensively analyze the role of HLA class I-restricted CD8+ T cell responses in developing pre-ART DRMs in different populations worldwide.

There are limitations to our study. The sample size of 266 participants is relatively small for examining over 1800 possible HLA by DRM associations. In addition, we did not compare the NGS results directly with the Sanger sequencing—a currently accepted standard assay. There may be differences in DRMs identified between the two assays. Although the pyrosequencing technology developed by 454 Life Sciences was the first NGS technology that provided high throughput and high-quality sequences. Roche has terminated support for the 454 FLX system since 2016 due to its relatively higher running cost and lower sequence output compared to other NGS platforms. Moreover, it is important to compare the DRMs identified in this ARV-naïve population with populations exposed to ART to evaluate the impact of pre-ART DRMs on the DRMs’ evolution under ART.

In conclusion, analysis of viruses from ART-naïve individuals in this study cohort identified high levels of HIV drug resistance to all classes of antiretroviral drugs (NNRTIs, NRTIs, PIs, and INSTIs) included in the current first-line ART regimens in Africa. The development of drug resistance may be influenced by immunological pressure exerted by HLA class I-restricted CTL responses. Our findings show that HLA genotyping may provide patients with better ARV drugs that are less likely to enhance DRMs’ development.

## Figures and Tables

**Table 1:** Characteristics of the study participants (*n* = 266).

Parameters	Median ± IQR *	*n*	%
Age (yrs)	35 ± 10	252	94.7
18–29		59	22.2
30–39		127	47.7
40+		66	24.8
Unknown		14	5.3
Country of origin		266	100
Kenya		126	47.4
Tanzania		123	46.2
Uganda		14	5.3
unknown		3	1.1
HIV Subtype		266	100
A1		150	56.4
B		1	0.4
C		14	5.3
D		25	9.4
G		1	0.4
AD		35	13.2
Other CRF		35	13.2
unknown		5	1.9
CD4+ T-cell count, cells/μL	259 ± 218.75	65	24.4
Duration of HIV-1 Positive (yrs)	7.67 ± 8.93	260	97.7

* IQR: interquartile rang (Q3–Q1); No: number; yrs: years.

**Table 2:** Drug class, population prevalence, and level of ARV drug resistance conferred of selected HIV-1 drug-resistant mutations found in the study participants.

		Level of ARV Drug Resistance Conferred by Mutation			DRM Represented in Databases		
Drug Class	Mutations (% T ^a^, D ^b^, L ^c^)	High-Level Resistance	Intermediate Level Resistance	Low-Level Resistance	IAS-USA	Stanford HIV Db	WHO Surveillance Mutation
NRTI	M184I * (37, 26, 11)	FTC, **3TC**			✓	✓	✓
	K65ER * (6, 1, 5)	**TDF**	**ABC**, FTC, **3TC**		✓	✓	✓
NNRTI	E138AK (15, 8, 7)		RPV		✓	✓	
	K101EN (5, 1,4)		**NVP**, RPV	DOR, **EFV**, ETR	✓	✓	✓
	G190ES (5, 2, 3)	DOR, **EFV**, **NVP**, RPV	ETR, DOR	RPV	✓	✓	✓
	L100IV (4, 0.4, 3.6)		**EFV**, **NVP**	RPV	✓	✓	✓
	K103NT (2, 0.5, 1.5)	**EFV**, **NVP**			✓	✓	✓
PI	D30N (10, 6, 4)	NFV ^‡^			✓	✓	✓
	M46I (8, 3, 5)			ATV/R, **LPV/r**	✓	✓	✓
	V82A (2, 0, 2)		**LPV/r**	ATV/r	✓	✓	✓
	N88S (1, 0, 1)	ATV/r			✓	✓	✓
	I47V (1, 0, 1)			**LPV/r**, DRV/r, ATV/r	✓	✓	✓
	I50V (0.5, 0, 0.5)	FPV	**LPV/R**	DRV/r	✓	✓	✓
INSTI	E138K ^†^ (8, 4, 4)			EVG, RAL, **DTG**, CAB, BIC	✓	✓	
	G118R (3, 2.6, 0.4)	CAB, EVG, **RAL**	**DTG**, BIC		✓	✓	
	R263K (3, 1, 2)		**DTG**, EVG, CAB, BIC	**RAL**	✓	✓	
	T66IA (2, 0, 2)	EVG		**RAL**	✓	✓	
	Y143CH (1.5, 0, 1.5)	**RAL**			✓	✓	

Mutations were selected based on the IAS-USA guidelines, Stanford HIV Db and WHO surveillance mutations [25,26]. ^a^ Total DRMs. ^b^ Dominant DRMs (>20%). ^c^ Low abundant DRMs (<20%). * HIV-1 DRM increases viral susceptibility to other NRTI medications (TDF, AZT). ^†^ Synergistic with other INSTI DRMs leading to intermediate level resistance. ^‡^ Discontinued medication. 3TC = Lamivudine, TDF = Tenofovir, FTC = Emtricitabine, ABC = Abacavir, RPV = Rilpivirine, NVP = Nevirapine, DOR = Doravirine, EFV = Efavirenz, ETR = Etravirine, NFV = Nelfinavir, ATV/r = Atazanavir, LPV/r = lopinavir, DRV/r = Darunavir, FPV = Fosamprenavir, EVG = Elvitegravir, RAL = Raltegravir, DTG = dolutegravir, CAB = Cabotegravir, BIC = Bictegravir. NRTI: nucleos(t)ide analogue reverse transcriptase inhibitor. NNRTI: non-nucleo(t)side analogue reverse transcriptase inhibitor. PI: protease inhibitor. INSTI: integrase strand transfer inhibitor. Bolded = recommended first line ARV drugs.

**Table 3:** Selected patient HLA allele and harbored HIV DRM positive associations.

DRM ^ (POPULATION PREVALENCE (% T ^A^, D ^B^, L ^C^)	HLA ^&^ ALLELE (HLA−DRM-, HLA+ DRM-, HLA- DRM+, HLA+ DRM+)	*p*-Value	Adjusted *p*-Value ^#^	Allele Frequency (>10%)	Allele Frequency (5–10%)
RT ^%^ E138K (11, 6, 5) RT G190ES (5, 2, 3)	A*66:01 (196,19,214,25)	0.038	1.000		Kenya, Uganda
	B*45:01 (202,15,18,7)	0.003	0.928	Rwanda, Uganda	Zimbabwe, Kenya, Cameroon, South African Black
	A*68:02 (201,29,3,4)	0.008	1.000	Kenya	Zimbabwe Ghana, South Africa, Uganda
PI ^§^ M46I (8, 3, 5)	C*04:05 (201,14,15,4)	0.046	1.000		
	C*04:07 (208,7,16,3)	0.038	1.000		
	C*17:01 (171,44,10,9)	0.018	1.000	Ghana, Kenya, South Africa Black	Zimbabwe, Uganda, South Africa
IN ^$^ T97A (12, 9, 3)	A*66:01 (200,13,14,12)	6.20 × 10^−7^	0.001		Kenya, Uganda
IN E138K (8, 4, 4)	A*68:01 (220,4,13,2)	0.048	1.000		Senegal
	A*68:02 (194,28,10,5)	0.042	1.000	Kenya	Zimbabwe Ghana, South Africa, Uganda
	B*15:17 (224,3,13,2)	0.032	1.000		
	B*35:02 (223,4,13,2)	0.043	1.000		
	C*17:01 (173,46,8,7)	0.021	1.000	Ghana, Kenya, South African Black	Kenya, Zimbabwe, Uganda, South Africa
IN R263K (3, 1, 2)	A*30:04 (225,8,4,2)	0.022	1.000		
	B*15:03 (200,36,2,4)	0.011	1.000		Kenya, Uganda, Zimbabwe, Senegal, South Africa

*p*-value was calculated by Fishers’s Exact Test, and less than 0.05 was considered significant. Associations were selected based on the clinical significance of the HIV DRM according to Stanford HIV Db [26]. ^ DRM: drug resistance mutation. ^A^ All DRMs ^B^ Dominant DRMs (>20%) ^C^ Low abundant DRMs (<20%) ^%^ RT: reverse transcriptase. ^§^ PI: protease inhibitor. ^$^ IN: integrase. “-” indicates the absence; “+” indicates the presence. ^&^ HLA: human leukocyte antigen. ^#^ Adjusted *p*-value was calculated from Fisher’s Exact Test, accounting for the 1856 comparisons between the identified DRMs and HLA alleles of the participants using the Benjamin–Hochberg method. *p*-value less than 0.05 was considered significant.

## Data Availability

Raw data is available upon request.

## Supplementary Materials

The following supporting information can be downloaded at: https://www.mdpi.com/article/10.3390/v14020273/s1, Table S1: Prevalence of selected HIV-1 drug resistant mutations among particpants from different countries.

## References

[1] (2021). The Global HIV/AIDS Epidemic, HIV.gov. https://www.hiv.gov/hiv-basics/overview/data-and-trends/global-statistics.

[2] UNAIDS, Update. 2020. Switzerland. Https://www.unaids.org/en/resources/documents/2020/global-aids-report.

[3] (2021). AIDS and the Sustainable Development Goals, UNAIDS. Switzerland. Https://www.unaids.org/en/AIDS_SDGs.

[4] Fast-Track–Strategy to End the AIDS Epidemic by 2030, UNAIDS. 2014. Switerland. Https://www.unaids.org/en/resources/documents/2014/JC2686_WAD2014report.

[5] Eisinger R.W., Dieffenbach C.W., Fauci A.S. (2019). HIV viral load and transmissibility of HIV infection. JAMA.

[6] Cohen M.S., Chen Y.Q., McCauley M., Gamble T., Hosseinipour M.C., Kumarasamy N., Hakim J.G., Kumwenda J., Grinsztejn B., Pilotto J.H. (2011). Prevention of HIV-1 infection with early antiretroviral therapy. N. Engl. J. Med..

[7] Rodger A.J., Cambiano V., Bruun T., Vernazza P., Collins S., Van Lunzen J., Corbelli G.M., Estrada V., Geretti A.M., Beloukas A. (2016). Sexual activity without condoms and risk of HIV transmission in serodifferent couples when the HIV-positive partner is using suppressive antiretroviral therapy. JAMA.

[8] Bavinton B.R., Pinto A.N., Phanuphak N., Grinsztejn B., Prestage G., Zablotska-Manos I.B., Jin F., Fairley C.K., Moore R., Roth N. (2018). Viral suppression and HIV transmission in serodiscordant male couples: An international, prospective, observational, cohort study. Lancet HIV.

[9] Clutter D.S., Jordan M.R., Bertagnolio S., Shafer R.W. (2016). HIV-1 drug resistance and resistance testing. Infect. Genet. Evol..

[10] World Health Organization HIV Drug Resistance. 2021. Switzerland. Https://www.who.int/publications/i/item/9789240039608.

[11] Fanales-Belasio E., Raimondo M., Suligoi B., Butto S. (2010). HIV virology and pathogenetic mechanisms of action: A brief overview. Ann. Ist. Super. Sanità.

[12] Brumme Z.L., Kinloch N.N., Sanche S., Wong A., Martin E., Cobarrubias K.D., Sandstrom P., Levett P.N., Harrigan P.R., Joy J.B. (2018). Extensive host immune adaptation in a concentrated North American HIV epidemic. AIDS.

[13] Gatanaga H., Murakoshi H., Hachiya A., Hayashida T., Chikata T., Ode H., Tsuchiya K., Sugiura W., Takiguchi M., Oka S. (2013). Naturally selected rilpivirine-resistant HIV-1 variants by host cellular immunity. Clin. Infect. Dis..

[14] Payne R., Muenchhoff M., Mann J., Roberts H.E., Matthews P., Adland E., Hempenstall A., Huang K.-H., Brockman M., Brumme Z. (2014). Impact of HLA-driven HIV adaptation on virulence in populations of high HIV seroprevalence. Proc. Natl. Acad. Sci. USA.

[15] Moore C.B., John M., James I.R., Christiansen F.T., Witt C.S., Mallal S.A. (2002). Evidence of HIV-1 adaptation to HLA-restricted immune responses at a population level. Science.

[16] Brumme Z., John M., Carlson J.M., Brumme C., Chan D., Brockman M., Swenson L., Tao I., Szeto S., Rosato P. (2009). HLA-Associated Immune Escape Pathways in HIV-1 Subtype B Gag, Pol and Nef Proteins. PLoS ONE.

[17] Gupta R.K., Gregson J., Parkin N., Haile-Selassie H., Tanuri A., Forero L.A., Kaleebu P., Watera C., Aghokeng A., Mutenda N. (2018). HIV-1 drug resistance before initiation or re-initiation of first-line antiretroviral therapy in low-income and middle-income countries: A systematic review and meta-regression analysis. Lancet Infect. Dis..

[18] Liang B., Murray M., Sampathkumar R., Luo M. (2020). HIV-1 genotypic drug resistance testing and Next-generation sequencing. Front. Clin. Drug Res.–HIV.

[19] WHO (2019). Update of Recommendations on First- and Second-Line Antiretroviral Regimens.

[20] Mbunkah H.A., Bertagnolio S., Hamers R.L., Hunt G., Inzaule S., De Wit T.F.R., Paredes R., Parkin N.T., Jordan M.R., Metzner K.J. (2020). Low-Abundance Drug-Resistant HIV-1 Variants in Antiretroviral Drug-Naive Individuals: A Systematic Review of Detection Methods, Prevalence, and Clinical Impact. J. Infect. Dis..

[21] Land A.M., Luo M., Pilon R., Sandstrom P., Embree J., Wachihi C., Kimani J., Plummer F.A., Ball T.B. (2008). High Prevalence of Genetically Similar HIV-1 Recombinants among Infected Sex Workers in Nairobi, Kenya. AIDS Res. Hum. Retrovir..

[22] Luo M., Blanchard J., Brunham K., Pan Y., Shen C.X., Lu H., Brunham R.C. (2001). Two-Step High Resolution Sequence-Based HLA-DRB Typing of Exon 2 DNA With Taxonomy-Based Sequence Analysis Allele Assignment. Hum. Immunol..

[23] Land A.M., Ball T.B., Luo M., Rutherford J., Sarna C., Wachihi C., Kimani J., Plummer F.A. (2008). Full-length HIV type 1 Proviral Sequencing of 10 Highly Exposed Women from Nairobi, Kenya Reveals a High Proportion of Intersubtype Recombinants. AIDS Res. Hum. Retrovir..

[24] Luo M., Blanchard J., Pan Y., Brunham K., Brunham R.C. (1999). High-resolution sequence typing of HLA-DQA1 and -DQB1 exon 2 DNA with taxonomy-based sequence analysis (TBSA) allele assignment. Tissue Antigens.

[25] Liu T.F., Shafer R.W. (2006). Web Resources for HIV Type 1 Genotypic-Resistance Test Interpretation. Clin. Infect. Dis..

[26] Wensing A.M., Calvez V., Ceccherini-Silberstein F., Charpentier C., Günthard H.F., Paredes R., Shafer R.W., Richman D.D. (2019). 2019 update of the drug resistance mutations in HIV-1. Top. Antivir. Med..

[27] IBM Corp (2020). IBM SPSS Statistics for Macintosh.

[28] National AIDS and STI Control Programme (2018). Guidelines on Use of Antiretroviral Drugs for Treating and Preventing HIV Infection in Kenya.

[29] Hassan A.S., Esbjörnsson J., Wahome E., Thiong’o A., Makau G.N., Price M.A., Sanders E.J. (2018). HIV-1 subtype diversity, transmission networks and transmitted drug resistance amongst acute and early infected MSM populations from Coastal Kenya. PLoS ONE.

[30] Gatanaga H., Brumme Z.L., Adland E., Reyes-Terán G., Avila-Rios S., Mejía-Villatoro C.R., Hayashida T., Chikata T., van Tran G., van Nguyen K. (2009). Potential for immune-driven viral polymorphisms to compromise antiretroviral-based pre-exposure prophylaxis for prevention of HIV-1 infection. AIDS.

[31] Crowell T.A., Danboise B., Parikh A., Esber A., Dear N., Coakley P., Kasembeli A., Maswai J., Khamadi S., Bahemana E. (2020). Pretreatment and Acquired Antiretroviral Drug Resistance Among Persons Living With HIV in Four African Countries. Clin. Infect. Dis..

[32] Hassan A.S., Bibby D.F., Mwaringa S.M., Agutu C.A., Ndirangu K.K., Sanders E.J., Cane P.A., Mbisa J., Berkley J.A. (2019). Presence, persistence and effects of pre-treatment HIV-1 drug resistance variants detected using next generation sequencing: A Retrospective longitudinal study from rural coastal Kenya. PLoS ONE.

[33] Crowell T.A., Kijak G.H., Sanders-Buell E., O’sullivan A.M., Kokogho A., Parker Z.F., Lawlor J., Polyak C.S., Adebajo S., Nowak R.G. (2019). Transmitted, pre-treatment and acquired antiretroviral drug resistance among men who have sex with men and transgender women living with HIV in Nigeria. Antivir. Ther..

[34] Li J.Z., Paredes R., Ribaudo H.J., Svarovskaia E.S., Metzner K.J., Kozal M.J., Hullsiek K.H., Balduin M., Jakobsen M.R., Geretti A.M. (2011). Low-frequency HIV-1 drug resistance mutations and risk of NNRTI-based antiretroviral treatment failure: A systematic review and pooled analysis. JAMA-J. Am. Med. Assoc..

[35] Li J.Z., Paredes R., Ribaudo H.J., Svarovskaia E.S., Kozal M., Hullsiek K.H., Miller M.D., Bangsberg D.R., Kuritzkes D.R. (2012). Relationship between minority nonnucleoside reverse transcriptase inhibitor resistance mutations, adherence, and the risk of virologic failure. AIDS.

[36] Beck I.A., Levine M., McGrath C.J., Bii S., Milne R.S., Kingoo J.M., So I., Andersen N., Dross S., Coombs R.W. (2020). Pre-treatment HIV-drug resistance associated with virologic outcome of first-line NNRTI-antiretroviral therapy: A cohort study in Kenya. EClinicalMedicine.

[37] Onywera H., Maman D., Inzaule S., Auma E., Were K., Fredrick H., Owiti P., Opollo V., Etard J.-F., Mukui I. (2017). Surveillance of HIV-1 pol transmitted drug resistance in acutely and recently infected antiretroviral drug-naïve persons in rural western Kenya. PLoS ONE.

[38] Makwaga O., Adhiambo M., Mulama D.H., Muoma J., Adungo F., Wanjiku H., Ongaya A., Maitha G.M., Mwau M. (2020). Prevalence of human immunodeficiency virus-1 drug-resistant mutations among adults on first- and second-line antiretroviral therapy in a resource-limited health facility in Busia County, Kenya. Pan Afr. Med. J..

[39] Kasang C., Kalluvya S., Majinge C., Stich A., Bodem J., Kongola G., Jacobs G.B., Mlewa M., Mildner M., Hensel I. (2011). HIV drug resistance (HIVDR) in antiretroviral therapy-naïve patients in Tanzania not eligible for WHO threshold HIVDR survey is dramatically high. PLoS ONE.

[40] Kulkarni R., Babaoglu K., Lansdon E.B., Rimsky L., van Eygen V., Picchio G., Svarovskaia E., Miller M.D., White K.L. (2012). The HIV-1 Reverse Transcriptase M184I Mutation Enhances the E138K-Associated Resistance to Rilpivirine and Decreases Viral Fitness. J. Acquir. Immune Defic. Syndr..

[41] Carlson J.M., Du V.Y., Pfeifer N., Bansal A., Tan V.Y., Power K., Brumme C., Kreimer A., DeZiel C.E., Fusi N. (2017). Impact of pre-Adapted HIV transmission. Nat. Med..

[42] McCluskey S.M., Kamelian K., Musinguzi N., Kigozi S., Boum Y., Bwana M.B., Muzoora C., Brumme Z.L., Carrington M., Carlson J. (2021). Pre-treatment integrase inhibitor resistance is uncommon in antiretroviral therapy-naive individuals with HIV-1 subtype A1 and D infections in Uganda. AIDS.

[43] Mueller S.M., Spriewald B.M., Bergmann S., Eismann K., Leykauf M., Korn K., Walter H., Schmidt B., Arnold M.-L., Harrer E.G. Influence of Major HIV-1 Protease Inhibitor Resistance Mutations on CTL Recognition. https://journals.lww.com/jaids.

