# Supplementary material for: High Level of Pre-Treatment HIV-1 Drug Resistance and Its Association with HLA Class I-Mediated Restriction in the Pumwani Sex Worker Cohort
Source: Viruses. 2022 Jan 28;14(2):273. doi: 10.3390/v14020273 (PMC8879707; doi:10.3390/v14020273)
Supplement: Supplementary file 1 [file viruses-14-00273-s001.zip › viruses-1524051-supplementary.pdf]

Table S1. Prevalence of selected HIV-1 drug resistant mutations among the participants from different countries.

Drug Class	DRMs	Prevalence (%)			*P* value *
		Kenya (n=126)	Tanzania (n=123)	Uganda (n=14)	
NNTI	M184I	40.2	43.9	28.6	0.7423
	K65R	4.7	11.4	7.1	0.7114
NNRTI	E138AK	18.1	7.3	7.1	0.1999
	K101EN	5.5	4.9	0.0	0.8304
	G190ES	7.1	6.5	0.0	0.8643
	K103E	75.6	78.8	85.7	0.7822
PI	D30N	7.8	13.8	14.3	0.3772
	M46I	6.3	12.2	16.7	0.2970
INSTI	E138K	6.3	8.1	7.1	0.8731
	T97A	12.6	15.4	28.6	0.4071

DRMs which prevalence is more than 5% in all participants were selected.

* *P* value is calculated based on chi-square test.
NRTI: nucleos(t)ide analogue reverse transcriptase inhibitor.
NNRTI: non-nucleo(t)side analogue reverse transcriptase inhibitor.
PI: protease inhibitor
INSTI: integrase strand transfer inhibitor.
